# A21 ROLE OF SIALIC ACID O-ACETYLATION IN MAINTAINING MUCUS INTEGRITY AND HOMEOSTASIS OF THE COLONIC MUCOSA

**DOI:** 10.1093/jcag/gwac036.021

**Published:** 2023-03-07

**Authors:** S Jafaripour, M Melvin, H Olsson, C Parrish, B Wasik, W Zandberg, K Bergstrom

**Affiliations:** 1 Biology, University of British Columbia - Okanagan, Kelowna, Canada; 2 Cornell Universty, Ithaca, United States; 3 Chemistry, University of British Columbia - Okanagan, Kelowna, Canada

## Abstract

**Background:**

The mucus network provides innate immune defense to protect our gastrointestinal tract from pathogens, and promote homeostasis with our resident microbiota. This network is constituted by the mucin MUC2 (Muc2 in mouse), which is ~80% complex O-linked glycans by weight. Sialic acid (Sia) is a key capping monosaccharide on complex O-glycans which has recently been linked to preserving mucus integrity. Sia can undergo enzymatic modifications including the addition of O-acetyl groups. The 9-O-acetyltransferase CasD1 is responsible for the 9-OAc Sia variants. Functionally, the OAc-modification is known to inhibit microbial sialidase activities which may preserve Sia’s protective roles on mucins. However, the extent of these OAc modifications in human and murine Mucin-2, and how they influence mucus function is unclear.

**Purpose:** To determine whether and how Sia O-acetylation on colonic mucus regulates mucus integrity, host-microbe interactions, and colitis susceptibility.

**Method:**

We used viral-derived probes that target specific OAc-Sia analogues on mucus on sections from human feces and mouse feces and colon tissues to visualize their spatial arrangement and microbial interaction in situ. For glycomics, OAc-Sia analogues were quantitated on purified human MUC2 and mouse Muc2 by HPLC-MS after derivatization with 4,5-dimethyl-1,2-diaminobenzamine (DMBA). O-glycans were released via non-reductive ammonia-catalyzed β-elimination and analyzed by mass spectrometry. For in vivo work, we generated intestinal epithelial cell-specific Casd1 KO mice (*Casd1*^flox/flox^;VillinCre or IEC *Casd1*^*-/*-^ mice) and analyzed their mucins. Sialidase activities were quantified in the supernatants of colon fecal materials from WT and IEC *Casd1*^*-/*-^ mutants mice using a fluorogenic substrate 4-MU-NeuNAc. Colitis susceptibility was monitored using 1.5% w/v Dextran Sodium Sulfate (DSS).

**Result(s):**

We found Sias on both human MUC2 and murine Muc2 were heavily O-acetylated, with ~75% and ~45% of Sias having 9-OAc-based modification in humans and mice respectively, and were distributed throughout the niche and barrier layers of mucus in situ. IEC *Casd1*^*-/*-^ mice were viable and healthy with knockdown confirmed by 9-OAc staining, western blot of protein lysates and mucins, and sialylomics. The mucus encapsulation appeared overall intact regardless of OAc status. However, IEC *Casd1*^*-/*-^ mice showed heightened susceptibility to 1.5% DSS colitis, linked to thinning of the mucus in IEC *Casd1*^*-/*-^ vs WT littermates after challenge. Consistent with the role of OAc Sia in sialidase inhibition, loss of OAc Sia was associated with increased sialidase activities as assessed by heightened 4 MU signal in fecal supernatants in WT vs littermate IEC *Casd1*^*-/*-^ mice. O-glycomics also showed reduction in the number of sialylated O-glycan structures upon loss of 9-OAc Sia.

**Conclusion(s):**

Sia O-acetylation appears important in maintaining key aspects of Sia-dependent mucus function and protecting from inflammatory insult.

**Please acknowledge all funding agencies by checking the applicable boxes below:** CCC

**Disclosure of Interest:**

None Declared

